# Supplementary figures and images for: Young children show negative emotions after failing to help others
Source: PLoS One. 2022 Apr 20;17(4):e0266539. doi: 10.1371/journal.pone.0266539 (PMC9020688; doi:10.1371/journal.pone.0266539)

**S1 Figure. An overview of the study set-up from a bird’s-eye perspective.**


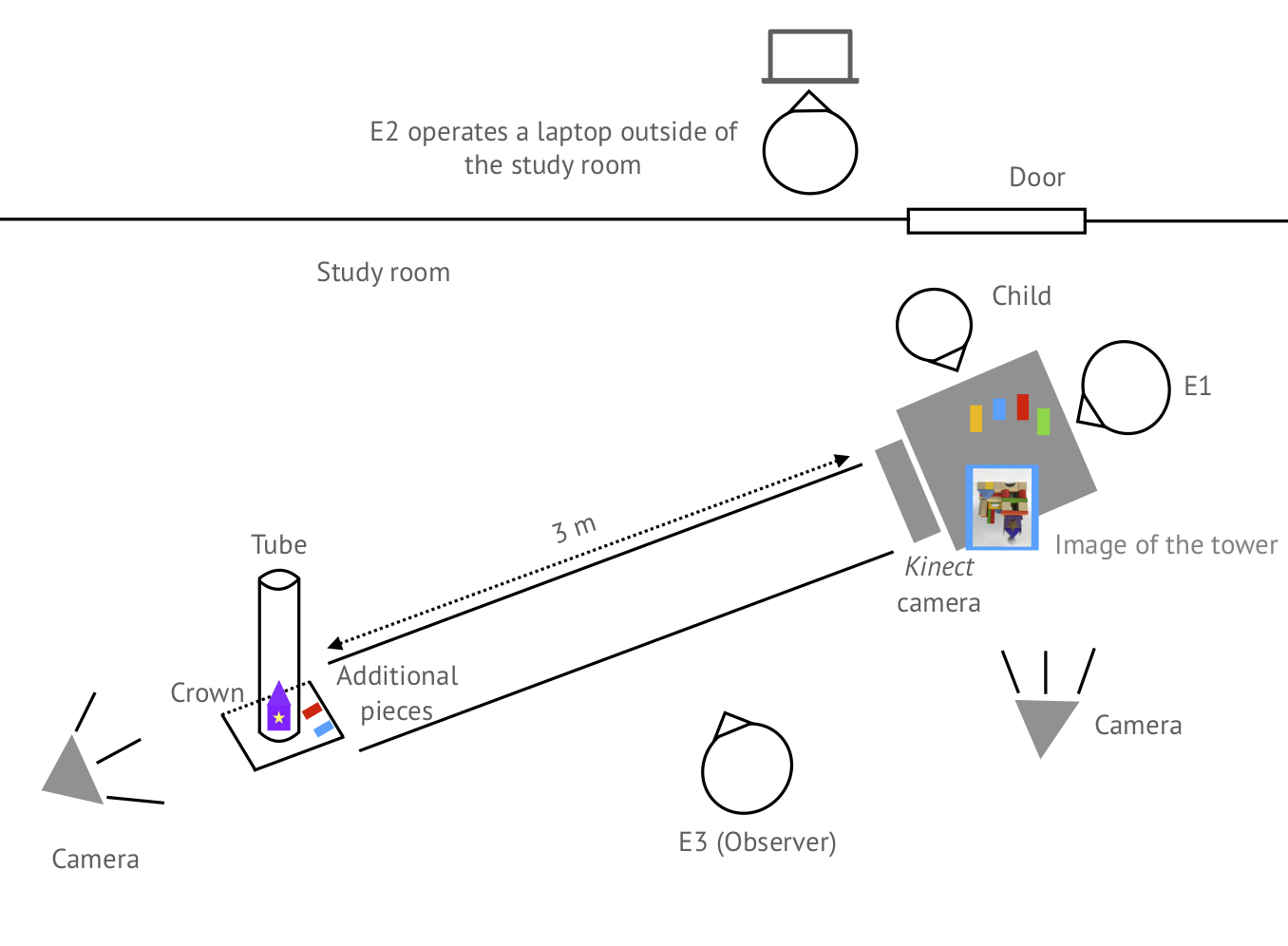

Supplement: S1 Fig — (DOCX) [file pone.0266539.s001.docx]
