# Supplementary material for: Young children show negative emotions after failing to help others
Source: PLoS One. 2022 Apr 20;17(4):e0266539. doi: 10.1371/journal.pone.0266539 (PMC9020688; doi:10.1371/journal.pone.0266539)
Supplement: S2 Fig — (DOCX) [file pone.0266539.s002.docx]

**S2 Figure. An illustration of the materials used in Studies 1 and 2.**

*(A) The Image of The Finished Tower that Children Were Shown During the Study. (B) The Tube Apparatus in Study 1. (C) The Chair Built by E3. (D) The Tube Apparatus in Study 2.*
