# Supplementary material for: Young children show negative emotions after failing to help others
Source: PLoS One. 2022 Apr 20;17(4):e0266539. doi: 10.1371/journal.pone.0266539 (PMC9020688; doi:10.1371/journal.pone.0266539)
Supplement: S1 Appendix — (DOCX) [file pone.0266539.s003.docx]

# S1 Appendix. Recording and pre-processing of body posture data (Study 1 and 2).

To record body posture data, the *Kinect* camera is controlled by a separate laptop with a script run in Matlab (Version 9.5). All recorded sequences provide body posture data for the location of x-, y- and z-coordinates of 20 skeletal points (see Figure 1 in the main manuscript). Recordings were processed using processing steps developed by Hepach et al. (2017). Several checks were conducted to ensure high data quality of the body posture data included in the analyses. For instance, the script ensured that children were walking upright, and their head was above the shoulders. An additional check ensured that children’s feet were crossed for each trial. If not, the skeleton was removed. In some cases, the issue emerged that children would run or jump and thus quickly move outside of the tracking range of the *Kinect*, which resulted in a substantial loss of data. Once valid walking sequences were selected, the values were binned and interpolated. The processed data comprised 20 data points per trial along 20 increments of children’s distance from the *Kinect* camera (see <https://tinyurl.com/y28pxcmb>). Increments of time-distance with less than 90% of the median number of data points were excluded from the analyses, resulting in 13 data points per trial. This approach parallels previous applications of body posture analyses (Hepach et al., 2017; Hepach & Tomasello, 2020).

**References**

Hepach, R., & Tomasello, M. (2020). Young children show positive emotions when seeing someone get the help they deserve. *Cognitive Development*, *56*, 100935. https://doi.org/10.1016/j.cogdev.2020.100935

Hepach, R., Vaish, A., & Tomasello, M. (2017). The fulfillment of others’ needs elevates children’s body posture. *Developmental Psychology*, *53*(1), 100–113. https://doi.org/10.1037/dev0000173
