# Supplementary material for: Young children show negative emotions after failing to help others
Source: PLoS One. 2022 Apr 20;17(4):e0266539. doi: 10.1371/journal.pone.0266539 (PMC9020688; doi:10.1371/journal.pone.0266539)
Supplement: S2 Appendix — (DOCX) [file pone.0266539.s004.docx]

# S2 Appendix. Additional details regarding the participants of Study 1.

**Table A**

The Number of Children whose Data was Excluded from Study 1

| Dropout Criterion |  |  | *N* | Details |  |
| --- | --- | --- | --- | --- | --- |
| 1) No data^1^ |  |  | 15 | The body posture pre-processing script did not result in any usable data for the respective child either on the first test trial or on all baseline trials (note: details regarding the pre-processing are provided through the online data repository) |  |
| 2) The child did not want to participate^1^ |  |  | 6 | The child became distressed during the study or indicated that she wanted to return to her kindergarten group during the study |  |
| 3) Apparatus error^1^ |  |  | 3 | The child was able to access the crown, because the tube was not adequately blocked by the plexiglass slate |  |
| 4) The child did not attempt to help^2,3^ |  |  | 13 | The child did not attempt to help or complete her own goal, i.e., the child did not interact with the plexiglass tube containing the crown during E1’s absence. |  |

*Notes.* The total number of excluded children for Study 1 was 37. We aimed for a sample size of 64 children, however, through technical improvements with regards to pre-processing, body posture data recorded by the *Kinect*, the final sample included 68 children.^1^ These exclusion criteria were pre-registered or are standard laboratory-wide exclusion criteria. ^2^This exclusion criterion was decided on after the beginning of the study. This situation did not occur during piloting. ^3^ There were two kinds of situations that occurred: 4.1) Children sometimes walked towards the tube and stood next to it but failed to interact with the tube during E1’s absence (N = 6). 4.2) In other cases, children remained close to the study table and did not walk towards the tube during E1’s absence (N = 7). This also meant that no body posture data could be recorded for the respective child.

**Table B**

*The Number (N) of Children Whose Data Was Excluded from the Analyses of Study 1 According to Condition and Drop-out Criterion (see Table A)*

| Dropout criterion |  | Help Observed | Help Unobserved | Own-Goal Observed | Own-Goal Unobserved |
| --- | --- | --- | --- | --- | --- |
| 1) |  | 1 | 5 | 2 | 7 |
| 2) |  | 2 | 0 | 0 | 4 |
| 3) |  | 0 | 0 | 1 | 2 |
| 4.1) |  | 3 | 0 | 3 | 0 |
| 4.2) |  | 0 | 5 | 0 | 2 |

**Table C**

The Number of Children who Provided Data on Each Trial and in Each Condition in Study 1

| Condition |  | *N* (Trial 1) | *N* (Trial 2) |
| --- | --- | --- | --- |
| Help Observed |  | 9F, 7M | 8F, 6M |
| Help Unobserved |  | 8F, 9M | 6F, 7M |
| Own-goal Observed |  | 9F, 8M | 9F, 7M |
| Own-goal Unobserved |  | 8F, 10M | 7F, 7M |

*Note.* M = Boys; F = girls
