# Supplementary material for: Young children show negative emotions after failing to help others
Source: PLoS One. 2022 Apr 20;17(4):e0266539. doi: 10.1371/journal.pone.0266539 (PMC9020688; doi:10.1371/journal.pone.0266539)
Supplement: S3 Appendix — (DOCX) [file pone.0266539.s005.docx]

## S3 Appendix. Detailed procedure of Study 1.

| Help observed | Help unobserved | Own-goal observed | Own-goal unobserved |
| --- | --- | --- | --- |
| Look, you can have a seat here. There’s a construction worker over there. He/she will watch you today *(E3 waves).* | Look you can have a seat here. | Look, you can have a seat here. There’s a construction worker over there. He/she will watch you today *(E3 waves).* | Look you can have a seat here. |
| I’ve just started to build my wonderful tower, and this is what it should look like at the end *(points to image)*. To build the tower, we must start with these building blocks here, and later we will have to get some additional blocks from back there *(points to tube).* And at the end my wonderful, hand-made crown needs to be placed on top *(points to image).* I’m really looking forward to completing my tower exactly like in this image, especially with my crown on it. *(In a sad voice)* But I would also be sad if my tower weren’t completed in the end. Should we start to build my tower? | | Look, you can build a tower today. This is what it should look like in the end *(points to image).* To build your tower, we must start with these building blocks here and later we will have to get some additional building blocks from back there *(points to tube)* And at the end this crown will have to be put on top *(points to image).* Without the crown your tower will not be completed. That’s why the crown must be placed on your tower in the end. Should we start to build your tower? | |
| *E1 engages children in the activity of building the tower. For instance, she asks: “where do you think this building block should go?” and gives the child the hint to look at the image, if the child does not guess correctly. Depending on goal context (help or own-goal), E1 consistently refers to the tower as either “my tower” or “your tower”.* | | | |
| Look, my crown *(points to image)* is covered in purple paper and has yellow stars on it. I made it myself, and I’m really looking forward to adding it to my tower in the end. *(In a sad voice)* But I would be sad if the crown weren’t added to my tower later. | | Look, the crown *(points to image)* is covered in purple paper and has yellow stars on it. It has to be placed on your tower at the end, so that it is completed. Because, without the crown, your tower will not be finished. | |
| Wow, my tower is almost finished! But in the end, my wonderful, hand-made crown needs to be added. Without the crown my tower would not be completed and *(in a sad voice)* I would be really sad, if my tower weren’t completed with my crown in the end. | | Wow, your tower is already almost finished! But, because we’ve already put in so much effort to build your tower, we have to finish it, especially by adding the crown in the end. Because, without the crown, your tower will not be finished. | |
| *(Once there are no more building blocks left)* Now we are still missing the red building block for *my/your* tower. Let’s go get it from back there *(points to tube).* Ok, let’s go see how the red block looks on *my/your* tower.  We are also still missing the blue building block. Let’s go get that one from back there. Let’s go see how the blue block looks on *my/your* tower *(E1 and the child walk to the tube and back to retrieve each block. While the child walks towards the table with the tower, two successive baseline recordings of the child’s body posture are taken.)* | | | |

| Great, my tower is almost completed. *(Looks at phone)* Oh no! It’s already so late! I have to leave now. But the construction worker will stay here *(points to E3)*. Oh no! My tower! It’s not finished. My wonderful, hand-made crown is still missing. *(In a sad voice)* I would be so sad if my tower weren’t completed with my crown. Could you help me to complete my tower and retrieve my crown for me? *(If the child nods or says yes)* Ok. When you hear a knock on the door later, you can come to the door, and then you will have to return to your group. *(E1 leaves the room).* | Great, my tower is almost completed. *(Looks at phone)* Oh no! It’s already so late! I have to leave now. Oh no! My tower! It’s not finished. My wonderful, hand-made crown is still missing. *(In a sad voice)* I would be so sad if my tower weren’t completed with my crown. Could you help me to complete my tower and retrieve my crown for me? *(If the child nods or says yes)* Ok. When you hear a knock on the door later, you can come to the door, and then you will have to return to your group. *(E1 leaves the room).* | Now your tower is almost completed. *(Looks at phone)* Oh no! It’s already so late! I have to leave now. But the construction worker will stay here *(points to E3)*. Oh no! Your tower! It’s not finished. The crown is still missing. Without the crown your tower is not complete. Could you complete your tower and retrieve the crown from the tube? *(If the child nods or says yes)* Ok. When you hear a knock on the door later, you can come to the door, and then you will have to return to your group. *(E1 leaves the room).* | Now your tower is almost completed. *(Looks at phone)* Oh no! It’s already so late! I have to leave now. Oh no! Your tower! It’s not finished. The crown is still missing. Without the crown your tower is not complete. Could you complete your tower and retrieve the crown from the tube? *(If the child nods or says yes)* Ok. When you hear a knock on the door later, you can come to the door, and then you will have to return to your group. *(afterwards E1 leaves the room).* |
| --- | --- | --- | --- |
| *Observed conditions: If the child asks E3 for help or for how to retrieve the crown, he/she answers: “I’m sorry I can’t help you” and “I don’t know how to get it”. All conditions: E1 knocks on the door after one minute and while the child walks towards the door the first test trial is recorded. (Once E1 is back in the room)* I just remembered that I completely forgot to remove the tape from the tube. It wasn’t possible for you to retrieve the crown because of that. That was my mistake! I came back to remove the tape, so you can retrieve the crown. *E1* r*emoves the tape from the tube and gives the crown to child. While the child walks to the tower to place the crown on the tower, the second test recording is taken in Study 1. In Study 2, the child is handed the crown after walking to the table.* So, now you can put *your/my* crown on the tower. Great! Now *your/my* tower is finished! *The child is given a sticker and returns to her kindergarten group.* | | | |
