# Supplementary material for: Young children show negative emotions after failing to help others
Source: PLoS One. 2022 Apr 20;17(4):e0266539. doi: 10.1371/journal.pone.0266539 (PMC9020688; doi:10.1371/journal.pone.0266539)
Supplement: S4 Appendix — (DOCX) [file pone.0266539.s006.docx]

# S4 Appendix. Additional body posture results of Study 1.

## Change in Chest Expansion

***First Trial Analysis***

Children’s chest expansion (in cm) decreased from baseline to the first test trial across all four conditions: help observed, 95% Confidence Interval (CI) [-1.68, -5.26]; help unobserved, 95% CI [-1.22, -4.94]; own-goal observed, 95% CI [-0.94, -5.49]; own goal unobserved, 95% CI [-1.72, -6.40] (see Figure 3 in the main manuscript). On the first test trial, the change in children’s chest expansion (children’s change in chest height corrected for their change in hip height) did not systematically vary as a function of the two-way interaction of observation and goal context, *χ*2(1) = 0, *p* = 1 (see Figure 3). There was also no three-way interaction of observation, goal context and distance, *χ*2(1) = 0.06, *p* = .8. The two-way interaction of observation and distance did not reach significance, *χ*2(1) = 3.32, *p* = .07. There was no two-way interaction of goal context and distance, *χ*2(1) = 0.68, *p* = .41. We did find that boys (*M* = -4.47, *SD* = 4.74) showed a greater decrease in chest expansion (in cm) than girls (*M* = -2.46, *SD* = 2.84), *χ*2(1) = 4.09, *p* = .04 (see Figure A). There was no effect of age, *χ*2(1) = 1.06, *p* = .3, on children’s change in chest expansion on the first trial.

***Omnibus Analysis (First and Second Trial)***

Children’s chest expansion (in cm) was no longer reliably below its baseline level across all four conditions on the second test trial: help observed, 95% CI, [0.37, -3.09]; help unobserved, 95% CI [0.43, -3.70]; own-goal observed, 95% CI [0.13, -3.79]; own goal unobserved, 95% CI [0.88, -3.83]. In the omnibus model predicting children’s change in chest expansion, there was no two-way interaction of observation and goal context, *χ*2(1) = 0, *p* = 1, nor a three-way interaction of observation, goal context and distance, *χ*2(1) = 0.15, *p* = .7. There was also no two-way interaction of goal context and distance, *χ*2(1) = 1.29, *p* = .26, nor of observation and distance, *χ*2(1) = 2.76, *p* = .1. The change in children’s chest expansion varied as a function of gender, *χ*2(1) = 6.92, *p* = .01. Averaging across both test trials, boys (*M* = -3.65, *SD* = 4.32) showed a greater decrease in chest expansion (in cm) than girls (*M* = -1.62, *SD* = 3.13). There was no effect of age, *χ*2(1) = 0.31, *p* = .58, on children’s change in chest expansion. In addition, there was an effect of trial, χ2(1) = 10.45, *p* = .001. Children’s chest expansion (in cm) was more reduced on the first (*M* = -3.46, *SD* = 4.01) than on the second test trial (*M* = -1.58, *SD* = 3.48).

**Table A**

*Results of the Model Predicting Children’s Change in Chest Expansion on the First Trial of Study 1 (Estimates and Standard Errors, Together with Confidence Limits, and Results of Likelihood-ratio Tests)*

| Term |  | Estimate | SE | CI lower | CI upper | *χ2* | DF | *P* |
| --- | --- | --- | --- | --- | --- | --- | --- | --- |
| (Intercept) |  | -0.035 | 0.012 | -0.059 | -0.012 |  |  | (1) |
| Observation |  | -0.003 | 0.013 | -0.028 | 0.024 |  |  | (1) |
| Context |  | -0.012 | 0.014 | -0.041 | 0.018 |  |  | (1) |
| z.distance(2, 3) |  | 0.007 | 0.003 | 0.001 | 0.012 |  |  | (1) |
| Gender |  | 0.018 | 0.009 | 0.002 | 0.034 | 4.09 | 1 | .04 |
| z.age(2) |  | 0.005 | 0.005 | -0.005 | 0.015 | 1.06 | 1 | .3 |
| Observation:Context |  | 0 | 0.018 | -0.037 | 0.035 | 0 | 1 | 1 |
| Context:z.distance |  | -0.003 | 0.003 | -0.009 | 0.003 | 0.68 | 1 | .41 |
| Observation:z.distance |  | -0.006 | 0.003 | -0.012 | 0 | 3.32 | 1 | .07 |

*Notes.* (1) Not indicated because of having limited interpretation. (2) Scaled to a mean of zero and standard deviation of 1. Reference levels were set as follows: Context = help goal context; Observation = observed; Gender = male. (3) The variable z. distance indicates children’s distance (standardized) from the Kinect.

**Table B**

*Results of the Omnibus Model Predicting Children’s Change in Chest Expansion Across the First and Second Test Trial of Study 1 (Estimates and Standard Errors, Together with Confidence Limits, and Results of Likelihood-ratio Tests)*

| Term |  | Estimate | SE | CI lower | CI upper | *χ2* | DF | *P* |
| --- | --- | --- | --- | --- | --- | --- | --- | --- |
| (Intercept) |  | -0.031 | 0.01 | -0.049 | -0.012 |  |  | (1) |
| Observation |  | -0.004 | 0.01 | -0.026 | 0.015 |  |  | (1) |
| Context |  | -0.006 | 0.012 | -0.029 | 0.017 |  |  | (1) |
| z.distance(2,3) |  | 0.007 | 0.002 | 0.002 | 0.012 |  |  | (1) |
| Gender |  | 0.02 | 0.007 | 0.005 | 0.033 | 6.92 | 1 | .01 |
| z.age(2) |  | 0.002 | 0.004 | -0.006 | 0.01 | 0.31 | 1 | .58 |
| z.trial(2) |  | 0.009 | 0.003 | 0.004 | 0.013 | 10.45 | 1 | .001 |
| Observation:Context |  | 0 | 0.015 | -0.03 | 0.031 | 0 | 1 | 1 |
| Context:z.distance |  | -0.003 | 0.003 | -0.009 | 0.002 | 1.29 | 1 | .26 |
| Observation:z.distance |  | -0.005 | 0.003 | -0.01 | 0.001 | 2.76 | 1 | .1 |

*Notes.* (1) Not indicated because of having limited interpretation. (2) Scaled to a mean of zero and standard deviation of 1. Reference levels were set as follows: Context = help goal context; Observation = observed; Gender = male. (3) The variable z.distance indicates children’s distance (standardized) from the Kinect.

**Figure A**

Boxplot Visualizing the Effect of Gender on the Average Change in Children’s Chest Expansion in Study 1. The Black Lines Inside the Boxes Represent Medians. The Lines above and Below the Median Mark the First and Fourth Quartile. The Whiskers Capture Extreme Observations and Black Dots Represent Observations that are 1.5 times the Interquartile Smaller than the First Quartile or Greater than the Fourth Quartile.

## Model Assumptions

After fitting the models, we checked whether the assumptions of a normal distribution of the residuals were fulfilled by visual inspection of a QQ-plot (Field et al., 2012) and a histogram of the residuals, which revealed no obvious deviation from normality of the residuals. In addition, homogeneity of variances was checked by plotting the residuals against fitted values, which revealed no obvious patterns of a change in variance depending on the fitted values (Quinn & Keough, 2002). However, the plot of the residuals against fitted values suggested that there may be influential cases. An examination of cook’s distance, using the package influence.ME (Nieuwenhuis et al., 2012), revealed this to not be the case as the maximum cook’s distance was < 1.

Collinearity, determined for a standard linear model lacking the random effects, appeared to be no issue (maximum Variance Inflation Factor: 1.06, Quinn & Keough 2002). We assessed model stability on the level of the estimated coefficients and standard de- viations by excluding the levels of the random effects one at a time (Nieuwenhuis et al., 2012), and using a function kindly provided by Roger Mundry. In addition, confidence intervals for estimates were calculated using the function bootMer() of the package *lme4*, and using a function kindly provided by Roger Mundry.

**Figure B**

*Diagnostic Plots for the First Trial Model Predicting Children’s Change in Chest Expansion in Study 1. The Top Left Panel Show the Residuals Plotted Against the Fitted Values. The Top Tight Panel Shows the QQ-Plot of the Residuals. The Bottom Left Panel Shows a Histogram of the Residuals.*

## Change in Chest Height

***First Trial Analysis***

In the preregistered model, on the first trial, the change in children’s chest height tended to vary as a function of the interaction of observation and goal context, *χ*2(1) = 2.96, *p* = .09 (see Figure C). There was no three-way interaction of observation, goal context and distance, *χ*2(1) = 0.54, *p* = .46. There were no additional two-way interactions of observation and distance, *χ*2(1) = 2.59, *p* = .11, or of goal context and distance, *χ*2(1) = 0.28, *p* = .6. Gender did not predict children’s change in chest height, *χ*2(1) = 0.12, *p* = .73. Yet, there was a weak influence of age, *χ*2(1) = 2.96, *p* = .09, on children’s change in chest height. Children’s chest height tended to decrease with age, β ± SE = -0.008 ± 0.005.

***Omnibus Analysis (First and Second Trial)***

In the omnibus model of children’s change in chest height, averaging across both test trials, the interaction of observation and goal context was significant, *χ*2(1) = 5.99, *p* = .01 (see Figure C). Averaging across both test trials, children showed a greater decrease in their chest height (in cm) in the help observed condition (*M* = -2.57, *SD* = 3.62) than in all other conditions (own-goal observed, *M* = -0.4, *SD* = 3.44; own-goal unobserved, *M* = -0.16, *SD* = 4.04; help unobserved, *M* = 0.22, *SD* = 3.15). There was no three-way interaction of observation, goal context and distance, *χ*2(1) = 0.19, *p* = .66. In addition, there was an interaction of observation and distance, *χ*2(1) = 4.82, *p* = .03, indicating that children’s posture in the observed condition increased in height as children walked towards the *Kinect*, β ± SE = 0.007 ± 0.003. This effect was not predicted, yet could be due to children elevating their posture as they stand up from kneeling down next to the tube. There was no interaction of goal context and distance, *χ*2(1) = 0.16, *p* = .69, or of gender, *χ*2(1) = 0.11, *p* = .73, on children’s change in chest height. Age had a significant impact on children’s change in chest height across both trials, *χ*2(1) = 5.96, *p* = .01. With age, children showed a greater decrease in their chest height, β ± SE = -0.008 ± 0.003. In addition, there was a clear effect of trial, *χ*2(1) = 12.72, *p* < .001. Children’s chest height (in cm) was more decreased on the first (*M* = -1.65, *SD* = 3.85), compared to the second test trial (*M* = 0.4, *SD* = 3.2).

## Change in Hip Height

***First Trial Analysis***

The change in children’s hip height was not clearly predicted by the interaction of observation and goal context on the first trial, *χ*2(1) = 1.96, *p* = .16. The three-way interaction of observation, goal context and distance was not significant, *χ*2(1) = 0.37, *p* = .54. There were no two-way interactions of observation and distance, *χ*2(1) = 0.11, *p* = .74, or of goal context and distance, *χ*2(1) = 0.01, *p* = .94. There was no effect of gender, *χ*2(1) = 2.51, *p* = .11, on children’s change in hip height. There was, however, an effect of age, *χ*2(1) = 5.32, *p* = .02. With age, children showed a greater decrease in their hip height, β ± SE = -0.012 ± 0.005.

***Omnibus Analysis (First and Second Trial)***

In the omnibus model, there was a trend towards an interaction observation and goal context, *χ*2(1) = 3.34, *p* = .07. Like children’s change in chest height, children’s hip height (in cm) tended to be more decreased in the help observed condition (*M* = -0.09, *SD* = 2.84) relative to all other conditions (own-goal observed, *M* = 2.15, *SD* = 3.47; own-goal unobserved, *M* = 2.77, *SD* = 5.6; help unobserved, *M* = 2.67, *SD* = 3.31). The three-way interaction of observation, goal context and distance was not significant, *χ*2(1) = 0.54, *p* = .46. There was no interaction of observation and distance, *χ*2(1) = 0.61, *p* = .44, or of goal context and distance, *χ*2(1) = 0.33, *p* = .57. There was an effect of gender on children’s change in hip height, *χ*2(1) = 4.82, *p* = .03. Averaging across both trials, boys (*M* = 2.95, *SD* = 4.47) showed a greater increase in hip height (in cm) than girls (*M* = 0.89, *SD* = 3.42). In addition, age had a significant impact on children’s change in hip height, *χ*2(1) = 5.29, *p* = .02. With age, children showed a greater decrease in their hip height averaging across both trials, β ± SE = -0.01 ± 0.004. There was no effect of trial on children’s change in hip height, *χ*2(1) = 0.4, *p* = .53.

In sum, in Study 1, averaging across both test trials, children’s chest height was most decreased in the help observed condition, consistent with our preregistered hypothesis (see Figure C). However, there was also a trend towards children’s hip height being most lowered in the help observed condition, suggesting that the results for children’s chest height may be due to a general decrease in children’s body posture in the help observed condition, rather than being specific to children’s upper body. Thus, we cannot conclude that the results for children’s change in chest height are not due to running, jumping, or crouching.

**Figure C**

Boxplots of the Average Change in Children’s Body Posture Depending on Observation and Goal Context on the First and Second Test Trial of Study 1. The Top Two Panels Show the Change in Children’s Chest Height. The Central Two Panels Show the Change in Children’s Hip Height. The Bottom Panels Show the Change in Children’s Chest Expansion (Change in Chest Height – Change in Hip Height). The Dashed Line Indicates the Baseline Level.

**References**

Field, A. P., Miles, J., & Field, Z. (2012). *Discovering statistics using R*. Sage.

Nieuwenhuis, R., te Grotenhuis, M., & Pelzer, B. (2012). Influence.ME: Tools for Detecting Influential Data in Mixed Effects Models. *The R Journal*, *4*, 10.

Quinn, G. P., & Keough, M. J. (2002). *Experimental Design and Data Analysis for Biologists.* Cambridge University Press.
