# Supplementary material for: Young children show negative emotions after failing to help others
Source: PLoS One. 2022 Apr 20;17(4):e0266539. doi: 10.1371/journal.pone.0266539 (PMC9020688; doi:10.1371/journal.pone.0266539)
Supplement: S5 Appendix — (DOCX) [file pone.0266539.s007.docx]

# S5 Appendix. Additional coding for Study 1.

## Text A. Duration of Children’s Attempt to Help or Complete Their Own Goal

To examine if children in all conditions of Study 1 spent an approximately equal amount of time attempting to retrieve the crown, a coder, blind to hypotheses and to the condition children were assigned to, coded the time children remained close to the tube from the point at which E1 left until her return. Inter-rater reliability was calculated based on approximately one quarter of the sample, which was coded by a second coder, also blind to hypotheses, *ICC* = .97 (*r* = .95). A Kruskall-Wallis H-test revealed that the unique combinations of observation and goal context did not predict the time children spent attempting to retrieve the crown, *H* = 5.74, *p* = .12. On average children remained close to the tube for slightly more than one minute (*M* = 65.19 s, *SD* = 11.32 s). 9% of children spent more than 1 SD shorter than the average (< 53.87 s) attempting to retrieve the crown.

## Text B. Emotion Valence Coding

***Method and Analysis***

We conducted a supplementary emotion valence coding to corroborate the validity of our body posture measures. Importantly, the body posture measures have already been partly validated as measures of emotion valence in prior work (Hepach et al., 2017, 2015; Hepach & Tomasello, 2020). However, given the novelty of our task, as well as of the exact measure (the change in children’s chest expansion as opposed to the change in children’s chest height), our aim was to provide further data to bear on the question whether the change in children’s upper body posture reflects changes in children’s emotion valence.

Two coders independently rated the valence of children’s emotional expression during the baseline phase, as well as on the first and second test trial on a scale from -4 (significantly negative) to +4 (significantly positive; see also Hepach et al., 2017 for a similar coding procedure). Ratings were conducted based on video stills (without audio), which corresponded exactly to those that were used to extract children’s body posture data (see Figure 1D in the main manuscript). Coders were instructed to look at all available video frames of a respective trial and base their emotion valence rating on the entire recording. The exact description for each of the codes was as follows: -4 = significantly negative, -3 = very negative, -2 = negative, -1 = slightly negative, 0 = neutral, 1 = slightly positive, 2 = positive, 3 = very positive, 4 = significantly positive. In addition, coders were asked to name children’s emotions in a free naming task, and to provide a reason for the name they chose and their valence rating. Coders were told to focus on children’s facial expression and body posture when providing a reason for the name they chose. For instance, one baseline rating read as follows: valence rating = 3, name = joyful, reason = big smile, looking straight, walking upright. We did not further analyze the emotion names or reason coders provided for their valence rating.

The emotion valence ratings for the first and second baseline trial were correlated with each other, *r* = .55. Therefore, the two ratings were averaged to create one baseline rating per child and coder. Across the baseline phase, and both test trials, inter-rater agreement was as follows: *ICC* = .58 (*r* = .49). Ratings were averaged across the two coders within each phase.

We examined the influence of phase (baseline, first trial or second trial) and the interactions of phase with observation and goal context on children’s emotion valence rating in linear models. Gender and age were included as control predictors. An additional analysis included the interaction of gender with phase because a pattern depending on gender was suggested by the change in children’s chest expansion. To test the significance of the predictors we compared a model that included the respective factor to one that did not include it yet included all other predictors.

***Results***

A full-reduced model comparison suggested a combined influence of phase, goal context and observation on children’s emotion valence, *F*(11, 179) = 30.61, *p* < .001, *r^2^* =.63. Follow-up analyses revealed no influence of the three-way interaction of phase, observation and goal context, *F*(2, 179) = 0.01, *p* = 0.99, nor the presence of any two-way interactions of phase with goal context or observation, *F*(5, 181) = 0.59, *p* = .71. There was also no two-way interaction of phase and gender, *F*(2, 184) = 0.08, *p* = .93.

There was only an overall influence of phase, *F*(2, 186) = 169.13, *p* < .001, on children’s emotion valence ratings. Children’s emotion valence was rated as more negative on the first trial (*M* = -1.34, *SD* = 1.29) relative to the baseline phase (*M* = 0.96, *SD =* 0.97), β ± SE = -2.29 ± 0.19, *t*(186) = -11.79, *p* < .001, and relative to the second test trial (*M* = 2.35, *SD* = 1.09), β ± SE = -3.68 ± 0.2, *t*(186) = -18.03, *p* < .001 (see Figure A). Moreover, children’s emotion valence on the second test trial was rated as more positive compared to baseline, β ± SE = 1.38 ± 0.20, *t*(186) = 6.79, *p* < .001.

***Conclusion***

In conclusion, children’s emotion valence varied as a function of phase, suggesting that children expressed a more negative emotional response immediately after failing to help, on the first test trial, than during the baseline phase and on the second test trial. This coding thus provides corroboration for our automatically recorded measure of the change in children’s upper body posture—children’s change in chest expansion—as an index of children’s emotional response (see also Hepach et al., 2015, 2017; Hepach & Tomasello, 2020).

**Figure A**

*Boxplot of Children's Rated Emotion Valence During the Baseline Phase, and on the First and Second Test Trial of Study 1. The Videos Used to Code Children’s Emotion Valence are the Same ones That Were Used to Automatically Extract Children’s Body Posture Data (see For Example Figure 1D). The Black Lines Inside the Boxes Represent Medians. The Lines above and Below the Median Mark the First and Fourth Quartile. The Whiskers Capture Extreme Observations and Black Dots Represent Observations that are 1.5 times the Interquartile Smaller than the First Quartile or Greater than the Fourth Quartile.*

**References**

Hepach, R., & Tomasello, M. (2020). Young children show positive emotions when seeing someone get the help they deserve. *Cognitive Development*, *56*, 100935. https://doi.org/10.1016/j.cogdev.2020.100935

Hepach, R., Vaish, A., & Tomasello, M. (2015). Novel paradigms to measure variability of behavior in early childhood: Posture, gaze, and pupil dilation. *Frontiers in Psychology*, *6*. https://doi.org/10.3389/fpsyg.2015.00858

Hepach, R., Vaish, A., & Tomasello, M. (2017). The fulfillment of others’ needs elevates children’s body posture. *Developmental Psychology*, *53*(1), 100–113. https://doi.org/10.1037/dev0000173
