# Supplementary material for: Young children show negative emotions after failing to help others
Source: PLoS One. 2022 Apr 20;17(4):e0266539. doi: 10.1371/journal.pone.0266539 (PMC9020688; doi:10.1371/journal.pone.0266539)
Supplement: S6 Appendix — (DOCX) [file pone.0266539.s008.docx]

# S6 Appendix. Additional details regarding the participants of Study 2.

**Table A**

*The Number of Children whose Data was not Included in Study 2*

| Dropout Criterion |  |  | *N* | Details |
| --- | --- | --- | --- | --- |
| 1) No data^1^ |  |  | 39 | The body posture pre-processing script did not result in any usable data for the respective child either on the first test trial or on all baseline trials (note: details regarding the pre-processing are provided through the online data repository) |
| 2) The child did not want to participate^1^ |  |  | 9 | The child became distressed during the study or indicated that she wanted to return to her kindergarten group during the study |
| 3) Apparatus error^1^ |  |  | 1 | The child was able to access the crown, because the tube was not adequately blocked by the plexiglass slate |
| 4) The child did not attempt to help^1,3^ |  |  | 9 | The child did not attempt to help or complete her own goal reach, i.e., the child did not interact with the plexiglass tube containing the crown during E1’s absence. |
| 5) Interruption of the study^2^ |  |  | 1 | The test session was interrupted by another Kindergarten group during the test phase |

*Notes.* The total number of children whose data was excluded from Study 2 was 59. ^1^ These exclusion criteria were pre-registered or are standard laboratory-wide exclusion criteria. ^2^This exclusion criterion was decided on after the beginning of the study. This situation did not occur during piloting. ^2^ There were two kinds of situations that occurred: 4.1) Children sometimes walked towards the tube and stood next to it but failed to interact with the tube during E1’s absence (N = 7). 4.2) In other cases, children remained close to the study table and did not walk towards the tube during E1’s absence (N = 2). This also meant that no body posture data could be recorded for the respective child.

**Table B**

*The Number (N) of Children Whose Data Was Excluded from the Analyses of Study 2 According to Age Group, Condition and Drop-out Criterion (see Table A)*

| Dropout criterion |  | Observed Age 5 | Unobserved Age 5 | Observed Age 4 | Unobserved Age 4 |
| --- | --- | --- | --- | --- | --- |
| 1) |  | 7 | 21 | 7 | 4 |
| 2) |  | 1 | 3 | 2 | 3 |
| 3) |  | 0 | 1 | 0 | 0 |
| 4.1) |  | 2 | 1 | 3 | 1 |
| 4.2) |  | 1 | 1 | 0 | 0 |
| 5) |  | 1 | 0 | 0 | 0 |

**Table C**

*The Number of Children who Provided Data on Each Trial, in Each Condition and Within Each Age Group in Study 2*

| Condition |  | *N* (Trial 1) | *N* (Trial 2) |
| --- | --- | --- | --- |
| Observed Age 5 |  | 11M, 17F | 7M, 16F |
| Observed Age 4 |  | 13M, 9F | 9M, 9F |
| Unobserved Age 5 |  | 9M, 8F | 8M, 5F |
| Unobserved Age 4 |  | 14M, 12F | 13M, 8F |

*Note.* M = Boys; F = girls
