# Supplementary material for: Young children show negative emotions after failing to help others
Source: PLoS One. 2022 Apr 20;17(4):e0266539. doi: 10.1371/journal.pone.0266539 (PMC9020688; doi:10.1371/journal.pone.0266539)
Supplement: S7 Appendix — (DOCX) [file pone.0266539.s009.docx]

## S7 Appendix. Additional details regarding the materials and procedure of Study 2.

## Text A. Materials for Study 2

The materials in Study 2 were identical to those used in Study 1, with one small change to the apparatus, with which we aimed to make the apparatus more approachable for the younger age group of children. As younger children showed a reluctance to interact with the tube apparatus during piloting, the crown was placed inside the plexiglass tube on top of a cylinder (see S2 Figure), which elevated the crown, so that it looked like it could be accessed even by 4-year-olds. Most children subsequently attempted to help by interacting with the tube.

## Text B. Procedure for Study 2

Two female research assistants took turns as the main experimenter (E1) and tested an approximately equal number of children in each combination of observation and age.

The procedure of Study 2 was nearly identical to the help context of Study 1 with two differences in the experimenters’ text, with which we aimed to make the procedure easier to follow and more motivating for the younger age group of children in Study 2. First, once children began building the tower with E1, children were praised for their helping, and referred to as helpers. E1 said: “It’s great that you are helping me to build my tower today. You really are a great helper”. We expected this prosocial identity attribution to increase young children’s motivation to help (e.g., Bryan et al., 2014). In addition, to slow the procedure down for the younger age group, in Study 2, E1 mentioned that she would be sad if her tower weren’t completed by the end of building it “because her tower would not be as pretty as in the picture”. Furthermore, the context in which the second test trial was recorded was altered. In Study 2, children did not carry the crown themselves, but rather walked beside E1 while she carried the tube towards the study table. This ensured that children’s posture on the second test trial was not altered because they were carrying an object (the crown).

**References**

Bryan, C. J., Master, A., & Walton, G. M. (2014). „Helping“ versus „being a helper“: Invoking the self to increase helping in young children. *Child Development*, *85*(5), 1836–1842. https://doi.org/10.1111/cdev.12244
