# Supplementary material for: Young children show negative emotions after failing to help others
Source: PLoS One. 2022 Apr 20;17(4):e0266539. doi: 10.1371/journal.pone.0266539 (PMC9020688; doi:10.1371/journal.pone.0266539)
Supplement: S8 Appendix — (DOCX) [file pone.0266539.s010.docx]

# S8 Appendix. Additional body posture results for Study 2.

## Change in Chest Expansion

***First Trial Analysis***

On the first test trial, 5-year-olds chest expansion (in cm) was below its baseline level in both the observed, 95% CI [-0.44, -2.71], and in the unobserved condition, 95% CI [-0.05, -2.40]. Four-year-olds’ chest expansion, however, did not reliably differ from baseline in either the observed, 95% CI [1.45, -0.75], or the unobserved condition, 95% CI [1.45, -1.82]. The model predicting the change in children’s chest expansion revealed no two-way interaction of observation and age, *χ*2(1) = 2.11, *p* = .15 (see Figure 5 in the main manuscript). There was also no three-way interaction of observation, age and distance, *χ*2(1) = 0.2 *p* = .66, nor were there two-way interactions of observation and distance, *χ*2(1) = 0.83, *p* = .36, or of age and distance, *χ*2(1) = 0.16, *p* = .69. In the final model, children’s change in chest expansion on the first trial did not vary as a function of the main effects of observation, *χ*2(1) = 0, *p* = .98; gender, *χ*2(1) = 0.01, *p* = .93, or distance, *χ*2(1) = 0, *p* = .99. The continuous age variable did not predict children’s change in chest expansion on the first test trial, *χ*2(1) = 0.81, *p* = .37. However, in an exploratory analysis, which included age as a factor (4- or 5-year-olds), children’s change in chest expansion on the first trial varied as a function of age, *χ*2(1) = 5.61, *p* = .02. 5-year-olds (*M* = -1.44, *SD* = 2.68) showed a more reduced chest expansion (in cm) than did 4-year-olds (*M* = 0.06, *SD* = 3.4).

***Omnibus Analysis (First and Second Trial)***

On the second test trial, neither 5- nor 4-year-olds’ chest expansion was reliably below its baseline level: 5-year-olds observed, 95% CI [0.94, -2.28]; 5-year-olds unobserved, 95% CI [0.73, -0.96]; 4-year-olds observed, 95% CI [1.26, -0.56] 4-year-olds unobserved, 95% CI [1.59, -0.97]. In the omnibus model, the change in children’s chest expansion was not predicted by the two-way interaction of observation and age, *χ*2(1) = 1.66, *p* = .2.

There was also no three-way interaction of observation, age, and distance, *χ*2(1) = 0.8, *p* = .37. There was neither an interaction of age and distance, *χ*2(1) = 0.11, *p* = .74, nor of observation and distance, *χ*2(1) = 0.41, *p* = .52. In the final model, including only main effects, children’s change in chest expansion was not predicted by observation, *χ*2(1) = 0, *p* = .96; gender, *χ*2(1) = 0.01, *p* = .92, or distance, *χ*2(1) = 0.12, *p* = .73. There was, however, an effect of trial, *χ*2(1) = 4.23, *p* = .04. Like in Study 1, children’s chest expansion (in cm) was more reduced on the first (*M* = -0.67, *SD* = 3.15) compared to the second test trial (*M* = -0.06, *SD* = 2.75). The continuous age variable did not predict children’s change in chest expansion in the omnibus model, *χ*2(1) = 1.12, *p* = .29. However, in the exploratory analysis, age was significant, *χ*2(1) = 4.59, *p* = .03. Averaging across both trials, 5-year-olds (*M* = -1.01, *SD* = 2.88) showed a more reduced chest expansion (in cm) than did 4-year-olds (*M* = 0.18, *SD* = 2.97).

**Table A**

*Results of the Model Predicting Children’s Change in Chest Expansion on the First Trial of Study 2 including Age as a Covariate (Estimates and Standard Errors, Upper and Lower 95% Confidence Limits, and Results of Likelihood-ratio Tests)*

| Term |  | Estimate | SE | CI lower | CI upper | χ2 | DF | *P* |
| --- | --- | --- | --- | --- | --- | --- | --- | --- |
| (Intercept) |  | -0.007 | 0.006 | -0.018 | 0.003 |  |  | (1) |
| z.age |  | -0.003 | 0.003 | -0.009 | 0.004 | 0.81 | 1 | .37 |
| Observation |  | 0 | 0.007 | -0.013 | 0.014 | 0 | 1 | .98 |
| Gender |  | 0.001 | 0.006 | -0.013 | 0.013 | 0.01 | 1 | .93 |
| z.distance(2) |  | 0 | 0.001 | -0.002 | 0.002 | 0 | 1 | .99 |

*Notes.* (1) Not indicated because of having limited interpretation. (2) Scaled to a mean of zero and standard deviation of 1. Reference levels were set as follows: Observation = observed, Gender = male. (3) The dependent variable is in meters.

**Table B**

*Results of the Model Predicting Children’s Change in Chest Expansion on the First Trial of Study 2 Including Age as a Factor (Estimates and Standard Errors, Upper and Lower 95% Confidence Limits, and Results of Likelihood-ratio Tests)*

| Term |  | Estimate | SE | CI lower | CI upper | χ2 | DF | *P* |
| --- | --- | --- | --- | --- | --- | --- | --- | --- |
| (Intercept) |  | 0 | 0.006 | -0.012 | 0.014 |  |  | (1) |
| Age |  | -0.015 | 0.006 | -0.028 | -0.003 | 5.61 | 1 | .02 |
| Observation |  | -0.001 | 0.006 | -0.013 | 0.011 | 0.03 | 1 | .86 |
| Gender |  | 0.002 | 0.006 | -0.011 | 0.015 | 0.11 | 1 | .74 |
| z.distance(2) |  | 0 | 0.001 | -0.002 | 0.002 | 0 | 1 | .99 |

*Notes.* (1) Not indicated because of having limited interpretation. (2) Scaled to a mean of zero and standard deviation of 1. Reference levels were set as follows: Observation = observed, Gender = male, Age = 4. (3) The dependent variable is in meters.

**Table C**

*Results of the Omnibus Model Predicting Children’s Change in Chest Expansion Across the First and Second Test Trial of Study 2 Including Age as a Covariate (Estimates and Standard Errors, Upper and Lower 95% Confidence Limits, and Results of Likelihood-ratio Tests)*

| Term |  | Estimate | SE | CI lower | CI upper | χ2 | DF | *P* |
| --- | --- | --- | --- | --- | --- | --- | --- | --- |
| (Intercept) |  | -0.004 | 0.005 | -0.014 | 0.005 |  |  | (1) |
| z.age(2) |  | -0.003 | 0.003 | -0.008 | 0.002 | 1.12 | 1 | .29 |
| z.distance(2) |  | 0 | 0.001 | -0.001 | 0.002 | 0 | 1 | .73 |
| Observation |  | 0 | 0.006 | -0.011 | 0.01 | 0.01 | 1 | .96 |
| Gender |  | 0.001 | 0.006 | -0.01 | 0.011 | 0.12 | 1 | .92 |
| z.trial(2) |  | 0.003 | 0.002 | 0 | 0.006 | 4.23 | 1 | .04 |

*Notes.* (1) Not indicated because of having limited interpretation. (2) Scaled to a mean of zero and standard deviation of 1. Reference levels were set as follows: Observation = observed, Gender = male. (3) The dependent variable is in meters.

**Table D**

*Results the Omnibus Model Predicting Children’s Change in Chest Expansion Across the First and Second Test Trial of Study 2 including Age as a Factor (Estimates and Standard Errors, Upper and Lower 95% Confidence Limits, and Results of Likelihood-ratio Tests)*

| Term |  | Estimate | SE | CI lower | CI upper | χ2 | DF | *P* |
| --- | --- | --- | --- | --- | --- | --- | --- | --- |
| (Intercept) |  | 0.001 | 0.005 | -0.009 | 0.013 |  |  | (1) |
| Age |  | -0.012 | 0.006 | -0.023 | -0.001 | 4.59 | 1 | .03 |
| Observation |  | 0 | 0.006 | -0.012 | 0.01 | 0.01 | 1 | .93 |
| Gender |  | 0.002 | 0.005 | -0.009 | 0.012 | 0.09 | 1 | .77 |
| z.distance(2) |  | 0 | 0.001 | -0.001 | 0.002 | 0.12 | 1 | .73 |
| z.trial(2) |  | 0.003 | 0.002 | 0 | 0.006 | 4.19 | 1 | .04 |

*Notes.* (1) Not indicated because of having limited interpretation. (2) Scaled to a mean of zero and standard deviation of 1. Reference levels were set as follows: Age = 4, Gender = male, Observation = observed. (3) The dependent variable is in meters.

## Model Assumptions

After fitting the models, we checked the model assumptions like in Study 1. Visual inspection of a QQ-plot (Field et al., 2012) and a histogram of the residuals revealed no obvious deviation from normality of the residuals. In addition, no obvious patterns of a change in variance depending on the fitted values was found (Quinn & Keough, 2002). The plot of the residuals against fitted values suggested that there may be influential cases. An examination of cook’s distance, using the package influence.ME (Nieuwenhuis et al., 2012), revealed this to not be the case as cook’s distance < 1.

Collinearity, determined for a standard linear model lacking the random effects, appeared to be no issue (maximum Variance Inflation Factor: 1.05, Quinn & Keough 2002). Model stability was estimated at the level of the estimated coefficients and standard de-viations by excluding the levels of the random effects one at a time (Nieuwenhuis et al., 2012), and using a function kindly provided by Roger Mundry.

**Figure A**

Diagnostic Plots for The First Trial Model Predicting Children’s Change in Chest Expansion in Study 2. The Top Left Panel Shows the Residuals Plotted Against the Fitted Values. The Bottom Left Panel Shows the QQ-Plot of the Residuals. The Top Right Panel Shows a Histogram of the Residuals.

## Change in Chest Height

***First Trial Analysis***

In preregistered analyses, on the first test trial, children’s change in chest height was not predicted by the two-way interaction of observation and age, *χ*2(1) = 1.85, *p* = .17 (see Figure B). There was also no three-way interaction of observation, age, and distance, *χ*2(1) = 0.43, *p* = .51. There were also no two-way interactions of age and distance, *χ*2(1) = 1.03, *p* = .31, or of observation and distance, *χ*2(1) = 0.68, *p* = .41. In the final model, comprising only main effects, children’s change in chest height did not vary as a function of observation, *χ*2(1) = 0.31, *p* = .58; age, *χ*2(1) = 0.32, *p* = .57; distance, *χ*2(1) = 0.05, *p* = .82, or gender, *χ*2(1) = 0.62, *p* = .43.

***Omnibus Analysis (First and Second Trial)***

The omnibus model, incorporating data from both test trials, revealed very similar results to the first trial model on children’s change in chest height. Children’s change in chest height across both trials was neither predicted by the two-way interaction of observation and age, *χ*2(1) = 1.46, *p* = .23, nor by the three-way interaction of observation, age, and distance, *χ*2(1) = 0.23, *p* = .63. Further, there were no effects of age and distance, *χ*2(1) = 0.59, *p* =.44, nor of observation and distance, *χ*2(1) = 1.59, *p* = .21. In the final model, there were no effects of observation, *χ*2(1) = 0.43, *p* = .51, or trial, *χ*2(1) = 1.07, *p* = .3, on children’s change in chest height. There were no additional effects of age, *χ*2(1) = 0.1, *p* = .75; distance, *χ*2(1) = 0, *p* = .97, or gender, *χ*2(1) = 1.1, *p* = .29.

## Change in Hip Height

***First Trial Analysis***

On the first trial, the change in children’s hip height, on the other hand, was predicted by the two-way interaction of observation and age, *χ*2(1) = 5.79, *p* = .02. Children’s hip height was more elevated with age in the observed condition, β ± SE = 0.019 ± 0.008 (see Figure B). There was no three-way interaction of observation, age, and distance, *χ*2(1) = .76, *p* = .38. In the final model, there was neither an interaction of age and distance, *χ*2(1) = 1.4, *p* = .24, nor of observation and distance, *χ*2(1) = 0.07, *p* = .8, and no effect of gender, *χ*2(1) = 0.05, *p* = .82.

***Omnibus Analysis (First and Second Trial)***

In the omnibus model, the change in children’s hip height was again predicted by the two-way interaction of observation and age, *χ*2(1) = 4.48, *p* = .03. Averaging across both trials, children’s hip height was more elevated with age in the observed condition, β ± SE = 0.016 ± 0.007 (see Figure B). There was no three-way interaction of observation, age, and distance, *χ*2(1) = 0.93, *p* = .33. There was no interaction of age and distance, *χ*2(1) = 0.83, *p* = .36, or of observation and distance, *χ*2(1) = 0.7, *p* = .4. No further effects of gender, *χ*2(1) = 0.4, *p* = .53, or trial, *χ*2(1) = 0.41, *p* = .52, were found.

In sum, in Study 2, we found that children with age showed an increased hip height both on the first test trial, and when averaging across the first and second test trial. As we had predicted that there would be no effects on children’s change in hip height, the effects on children’s change in chest height (without correcting children’s change in chest height for the change in children’s hip height) are difficult to interpret. Therefore, we interpret only the corrected estimate of children’s change in chest height (i.e., children’s change in chest expansion) here.

**Figure B**

Boxplots of the Average Change in Children’s Body posture Depending on Observation and Age on the First and Second Test Trial of Study 2. The Top Two Panels Show the Change in Children’s Chest Height. The Central Two Panels Show the Change in Children’s Hip Height. The Bottom Panels Show the Change in Children’s Chest Expansion (Change in Chest Height – Change in Hip Height). The Dashed Line Indicates the Baseline Level.

**References**

Field, A. P., Miles, J., & Field, Z. (2012). *Discovering statistics using R*. Sage.

Nieuwenhuis, R., te Grotenhuis, M., & Pelzer, B. (2012). Influence.ME: Tools for Detecting Influential Data in Mixed Effects Models. *The R Journal*, *4*, 10.

Quinn, G. P., & Keough, M. J. (2002). *Experimental Design and Data Analysis for Biologists.* Cambridge University Press.
