# Supplementary material for: Young children show negative emotions after failing to help others
Source: PLoS One. 2022 Apr 20;17(4):e0266539. doi: 10.1371/journal.pone.0266539 (PMC9020688; doi:10.1371/journal.pone.0266539)
Supplement: S9 Appendix — (DOCX) [file pone.0266539.s011.docx]

# S9 Appendix. Additional coding for Study 2.

## Text A. Duration of Children’s Attempt to Help

The duration of children’s attempt to help was coded like in Study 1. Inter-rater reliability was established for 25% of the data, ICC = .90 (r = .83). A Kruskal-Wallis H-test revealed that the time children spent attempting to help across the four combinations of age and observation did not differ significantly, *H* = 1.76, *p* = .62. On average, children remained close to the tube for almost the whole minute that E1 spent outside of the study room (*M* = 55.41 s, *SD* = 15.93 s). 16% of the children spent more than 1 SD less than the average (< 39.48 s) attempting to retrieve the crown.

## Text B. Discrete Emotion Coding

***Background, Method and Analysis***

We conducted an additional emotion coding with the aim of identifying the discrete emotions that were elicited by children’s failed attempt to help and correlating children’s emotion ratings with their change in their body posture. Our aim with this emotion coding was to assess if children predominantly expressed a negative self-conscious emotion after failing to help. Two coders, blind to the study hypotheses, independently rated the presence of four emotions, namely, shame, anger, sadness, and happiness on a scale from 1 to 5 with 1 = “does not show this emotion at all”, 3 = “shows this emotion a little bit” and 5 = “shows this emotion very much” on the first test trial (Over et al., 2016). Ratings were conducted based on video stills (without audio) of the *Kinect* recordings (see Figure 1D in the main manuscript). Coders were instructed to watch all the available video frames of the first test trial and provide an overall emotion rating for the entire recording. These video stills were the same ones that were used to extract children’s body posture data. Coders were provided a feature-based description of each emotion, and were instructed, in addition, to rely on their naïve intuition regarding what these emotions look like. This coding represents an improvement over the emotion coding of Study 1, because coders rated children’s emotional response based on a set of objectifiable features. More specifically, we provided coders established cues to identify shame (an averted or downturned gaze, a lowered upper body posture, frowning, and a similarity to embarrassment; Lewis et al., 1992; Stipek et al., 1992; Witkower & Tracy, 2019). Coders were also provided with established cues to identify the three other emotions (see Table A). Inter-rater agreement was as follows: shame, *ICC* = .73 (*r* = .59), sadness, *ICC* = .79 (*r* = .67), anger*, ICC* = .43 (*r* = .34), and happiness, *ICC* = .88 (*r* = .78). We did not include guilt in our coding scheme, because guilt has been argued to have no clear nonverbal emotion display (Keltner & Buswell, 1996; Vaish, 2018).

***Results***

An omnibus Friedman test indicated that the type of emotion predicted children’s average emotion rating on the first test trial, *χ*^2^(2) = 62.7, *p* < .001. We conducted follow-up focused comparisons using Wilcoxon signed rank tests with a Bonferroni-corrected alpha-level (α = 0.05/6 = .0083). These analyses showed that children’s emotional response was rated as more shame-like than like sadness, *T =* 639, *p* < .001, 95% CI [-0.75, -0.25], *d* = 0.39; happiness, *T =* 475, *p* < .001, [-1.5, -1], *d* = 1.19; and anger, *T =* 104.5, *p* < .001, [-1.5, -1], *d* = 1.46. Similarly, children’s emotional expression on the first test trial was rated as more like sadness than happiness, *T =* 2410.5, *p* < .001, [-1, -0.75], *d* = 0.9; and anger, *T =* 323, *p* < .001, [-1, -0.75], *d* = 1.21. There was no clear difference between children’s ratings of happiness and anger, *T =* 499.5, *p* = .84, [-0.75, 0.50], *d* = -0.14 (see Table B).

Children’s rating of happiness, moreover, was correlated with their change in chest expansion, spearman’s *rho*  = .25, *p* = .01, on the first trial. None of the other emotion ratings for negative emotions (shame, sadness, or anger) showed a similarly clear association with the change in children’s chest expansion (see Table C). None of the emotion ratings was clearly associated with children’s change in chest height.

**Table** **A**

*Emotion Descriptions Provided to Coders for the Discrete Emotion Coding of Study 2*

| Emotion |  | Description |
| --- | --- | --- |
| Sadness |  | Sad facial expression (frowning and furrowed brow)^1,2^, head tilted down^3^, slightly lowered body posture^3^ |
| Shame |  | Lowered upper body posture^3,4,5^, slumped shoulders^3,4^, frowning^5^, lowered head and/or lowered or avoidant gaze^3,4,5^, like embarrassment^3^ |
| Anger |  | Angry facial expression (tightened eyelids, furrowed brows and lips pressed together)^1,2^, large movements (e.g., a lot of arm movement and a heavy-footed gait)^3^, fists clenching^3^ |
| Happiness |  | Smile^1,2^, Head tilted upward^3^ |

*Notes.* The respective emotion features are based on the following references: ^1^Ekman & Friesen, 1978; ^2^Du et al., 2014; ^3^Witkower & Tracy, 2019; ^4^Lewis et al., 1992; ^5^Stipek et al., 1992

**Table B**

*Descriptive Statistics and the Results of Pairwise Comparisons for the Discrete Emotion Coding of Study 2*

| Emotion |  | *M* (*SD*) |
| --- | --- | --- |
| Shame |  | 2.19 (0.87)_abd_ |
| Sadness |  | 1.88 (0.68)_ab_ |
| Anger |  | 1.20 (0.41)_a_ |
| Happiness |  | 1.28 (0.66)_a_ |

*Notes*. Means with different subscripts differ from each other at *p* < .001. Ratings were conducted on a scale from 1 = “does not show this emotion at all”, 3 = “shows this emotion a little bit” to 5 = “shows this emotion a lot”. Note that the ratings for shame, anger, and sadness were conducted such that higher ratings indicate more negative emotions, while for happiness higher ratings indicate a more positive emotion.

**Table C**

*Spearman’s Rank-Order Correlations of the Discrete Emotion Coding with Children’s Change in Chest Height, Change in Hip Height and Change in Chest Expansion in Study 2*

| Emotion |  | Correlation Chest Height | | |  | Correlation Hip Height | |  | Correlation Chest Expansion | |
| --- | --- | --- | --- | --- | --- | --- | --- | --- | --- | --- |
|  |  | *rho* | | *p* |  | *rho* | *p* |  | *rho* | *p* |
| Shame |  | .05 | | .63 |  | .17 | .11 |  | -.15 | .14 |
| Sadness |  | .06 | .59 | |  | .12 | .26 |  | -.09 | .39 |
| Anger |  | -.1 | .2 | |  | .13 | .45 |  | -.25 | .07 |
| Happiness |  | -.06 | .59 | |  | -.29 | .01 |  | .25 | .01 |

## Text C. Feature-based Emotion Coding

We also conducted an emotion coding, in which coders rated the occurrence of several emotion features indicative of a negative self-conscious emotion separately (head/gaze aversion, lowered body posture, slumped shoulders and frowning) based on video recordings of the first test trial. Yet, on average, the reliability for this coding was poor (range of kappas: κ = .28 to .33). Coders explained that they had difficulty separating features such as a lowered head or gaze from other body posture features. Hence, we decided not to further analyze these data. The raw data for the feature-based coding is provided in the online data repository.

## Text D. Language Coding

***Background, Method and Analysis***

Children’s language was transcribed and coded while they attempted to retrieve the crown and shortly thereafter in Study 2. With this coding, our aim was to determine whether children showed evidence of self-evaluation. (e.g., “I am no good at this” see Lewis et al., 1992; Stipek et al., 1992). In our study, however, children did not frequently utter sentences like the one above. Rather children frequently described the situation, for instance by saying “I cannot reach the crown”. These sentences, while perhaps implying self-evaluation, may also be evaluatively neutral. We nonetheless provide results on children’s I-statements (statements children made about their ability to reach the crown). Coders scored whether children uttered at least one such I-statement (=1) or not (=0) while attempting to retrieve the crown of shortly thereafter. Inter-rater agreement established for 25% of the data was as follows: κ = .92. Coders also established whether children showed an I-statement in the presence of E1 or after she returned to the study room, towards the observer (E3) or while they were alone.

***Results***

Table D illustrates that 4- and 5-year-old children showed *I*-statements in both conditions. To examine whether the frequency of children’s *I*-statements differed depending on observation, age, or gender, we used generalized linear models with a binomial response term. There was no interaction of observation age, β *±* SE = 0.21 ± 0.95*, z* = 0.22, *p* = .82. Therefore, the final model included only main effects. The final model revealed that neither observation, β *±* SE = -0.61 *±* 0.47, *z* = -1.29, *p* = .2, nor age, β *±* SE = -0.32 *±* 0.47, *z* = -0.68, *p* = .5, clearly predicted children’s the probability to utter an *I*-statements. There was a marginal effect of gender, β ± SE = 0.86 *±* 0.47, *z* = 1.84, *p* = .07. A slightly higher proportion of girls (41%) than boys (23%) uttered an *I*-statement. Although children in the unobserved condition had fewer opportunities to speak, *I*-statements still occurred at about equal rates in both conditions.

Table D further illustrates that whom children showed spoke to differed depending on condition. Children in the unobserved condition spoke primarily with E1 after her return and/or to themselves, while children in the observed condition primarily spoke with the observer and/or with E1.

**Table D**

*Proportion and Number (N) of Children Who Showed an I-Statement Depending on Observation and Age. Columns to the Right show the Number of Children Who Showed an I-Statement Towards each Recipient.*

| Observation and Age |  | Prop. (*N*) | To E3 | To E1 | To self |
| --- | --- | --- | --- | --- | --- |
| Observed 4-year-olds |  | .41 (9) | 9 | 4 | - |
| Observed 5-year-olds |  | .36 (10) | 9 | 1 | - |
| Unobserved 4-year-olds |  | .27 (7) | - | 6 | 3 |
| Unobserved 5-year-olds |  | .24 (4) | - | 4 | 1 |
| Total |  | .32 (31) | 18 | 15 | 4 |

*Note.* Children could speak to both E3 (the observer) and E1 or to themselves and E1.

**References**

Du, S., Tao, Y., & Martinez, A. M. (2014). Compound facial expressions of emotion. *Proceedings of the National Academy of Sciences*, *111*(15), E1454–E1462. https://doi.org/10.1073/pnas.1322355111

Ekman, P., Friesen, W. V., & Hager, J. (1978). *Facial action coding system: A technique for the measurement of facial movement*. Consulting Psychologists Press.

Keltner, D., & Buswell, B. N. (1996). Evidence for the distinctness of embarrassment, shame, and guilt: A study of recalled antecedents and facial expressions of emotion. *Cognition and Emotion*, *10*(2), 155–172. https://doi.org/10.1080/026999396380312

Lewis, M., Alessandri, S. M., & Sullivan, M. W. (1992). Differences in Shame and Pride as a Function of Children’s Gender and Task Difficulty. *Child Development*, *63*(3), 630–638. JSTOR. https://doi.org/10.2307/1131351

Over, H., Vaish, A., & Tomasello, M. (2016). Do young children accept responsibility for the negative actions of ingroup members? *Cognitive Development*, *40*, 24–32. https://doi.org/10.1016/j.cogdev.2016.08.004

Stipek, D., Recchia, S., McClintic, S., & Lewis, M. (1992). Self-Evaluation in Young Children. *Monographs of the Society for Research in Child Development*, *57*(1), i–95. https://doi.org/10.2307/1166190

Vaish, A. (2018). The prosocial functions of early social emotions: The case of guilt. *Current Opinion in Psychology*, *20*, 25–29. https://doi.org/10.1016/j.copsyc.2017.08.008

Witkower, Z., & Tracy, J. L. (2019). Bodily Communication of Emotion: Evidence for Extrafacial Behavioral Expressions and Available Coding Systems: *Emotion Review*, *11*(2), 184–193. https://doi.org/10.1177/1754073917749880
